# Association of ambient air pollution with cardiovascular disease risks in people with type 2 diabetes: a Bayesian spatial survival analysis

**DOI:** 10.1186/s12940-020-00664-0

**Published:** 2020-11-05

**Authors:** Pei-Fang Su, Fei-Ci Sie, Chun-Ting Yang, Yu-Lin Mau, Shihchen Kuo, Huang-Tz Ou

**Affiliations:** 1 Department of Statistics, National Cheng Kung University, Tainan, Taiwan; 2 Institute of Clinical Pharmacy and Pharmaceutical Sciences, College of Medicine, National Cheng Kung University, 1 University Road, Tainan, 701 Taiwan; 3 Division of Metabolism, Endocrinology & Diabetes, Department of Internal Medicine, University of Michigan Medical School, Ann Arbor, MI USA; 4 Department of Pharmacy, College of Medicine, National Cheng Kung University, Tainan, Taiwan; 5 Department of Pharmacy, National Cheng Kung University Hospital, Tainan, Taiwan

**Keywords:** Time-to-event, Survival, Spatial correlation, Bayesian approach, Cardiovascular disease, Type 2 diabetes

## Abstract

**Background:**

Evidence is limited on excess risks of cardiovascular diseases (CVDs) associated with ambient air pollution in diabetic populations. Survival analyses without considering the spatial structure and possible spatial correlations in health and environmental data may affect the precision of estimation of adverse environmental pollution effects. We assessed the association between air pollution and CVDs in type 2 diabetes through a Bayesian spatial survival approach.

**Methods:**

Taiwan’s national-level health claims and air pollution databases were utilized. Fine individual-level latitude and longitude were used to determine pollution exposure. The exponential spatial correlation between air pollution and CVDs was analyzed in our Bayesian model compared to traditional Weibull and Cox models.

**Results:**

There were 2072 diabetic patients included in analyses. PM_2.5_ and SO_2_ were significant CVD risk factors in our Bayesian model, but such associations were attenuated or underestimated in traditional models; adjusted hazard ratio (HR) and 95% credible interval (CrI) or confidence interval (CI) of CVDs for a 1 μg/m^3^ increase in the monthly PM_2.5_ concentration for our model, the Weibull and Cox models was 1.040 (1.004–1.073), 0.994 (0.984–1.004), and 0.994 (0.984–1.004), respectively. With a 1 ppb increase in the monthly SO_2_ concentration, adjusted HR (95% CrI or CI) was 1.886 (1.642–2.113), 1.092 (1.022–1.168), and 1.091 (1.021–1.166) for these models, respectively.

**Conclusions:**

Against traditional non-spatial analyses, our Bayesian spatial survival model enhances the assessment precision for environmental research with spatial survival data to reveal significant adverse cardiovascular effects of air pollution among vulnerable diabetic patients.

**Graphical abstract:**

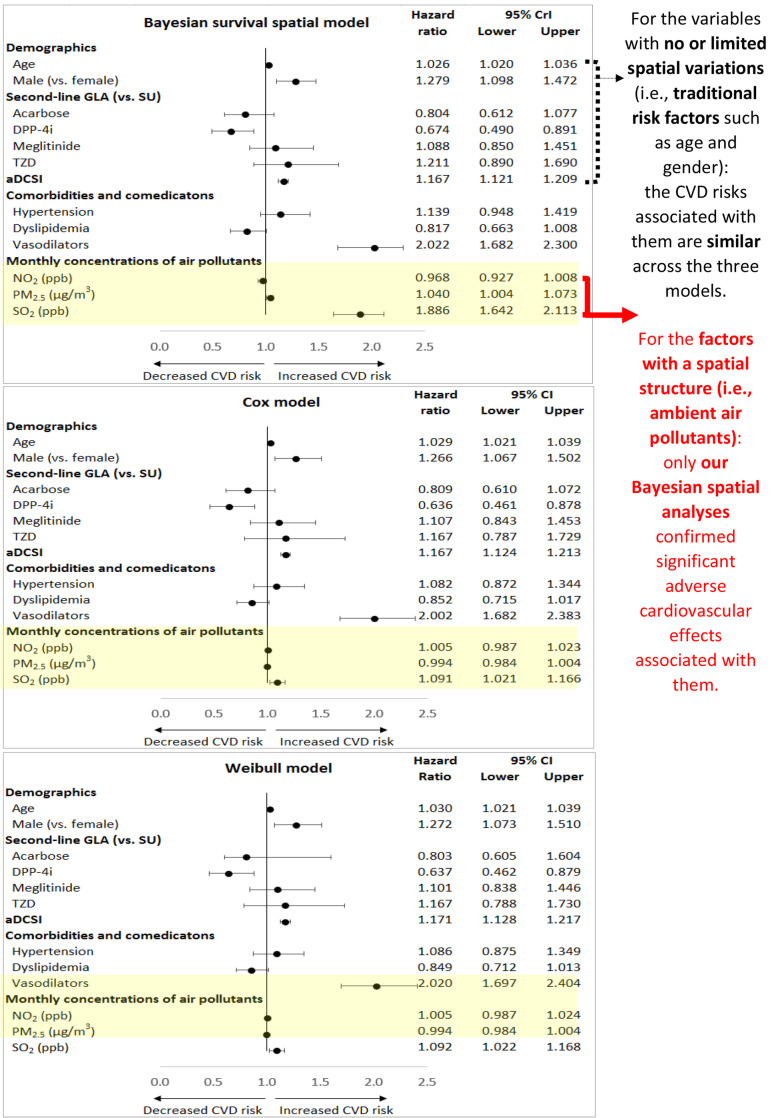

**Supplementary information:**

**Supplementary information** accompanies this paper at 10.1186/s12940-020-00664-0.

## Introduction

Mounting evidence indicates that elevated exposure to air pollution has been linked to increased risks of cardiovascular diseases (CVDs) [1–9] and reduction in the ambient PM_2.5_ concentrations may be associated with improved life expectancy [10]. Existing studies on adverse effects of air pollution typically consider the general population, while this issue is poorly understood among more vulnerable patients such as those with diabetes, who have a disproportionately greater risk of developing CVDs (a 1- to 3-fold higher risk for men and a 2- to 5-fold higher risk for women [11]) and fatal CVD death (1.7 times higher risk [5]) compared to the general population. Supporting evidence indicates that type 2 diabetes (T2D) patients are vulnerable to environmental hazards [12, 13]. However, compared to substantial evidence of adverse cardiovascular effects of air pollution in the general population, research on this topic for T2D population is in a great demand, particularly for those with established CVDs who are more vulnerable to deleterious cardiovascular outcomes [14, 15].

**Fig. 1:**
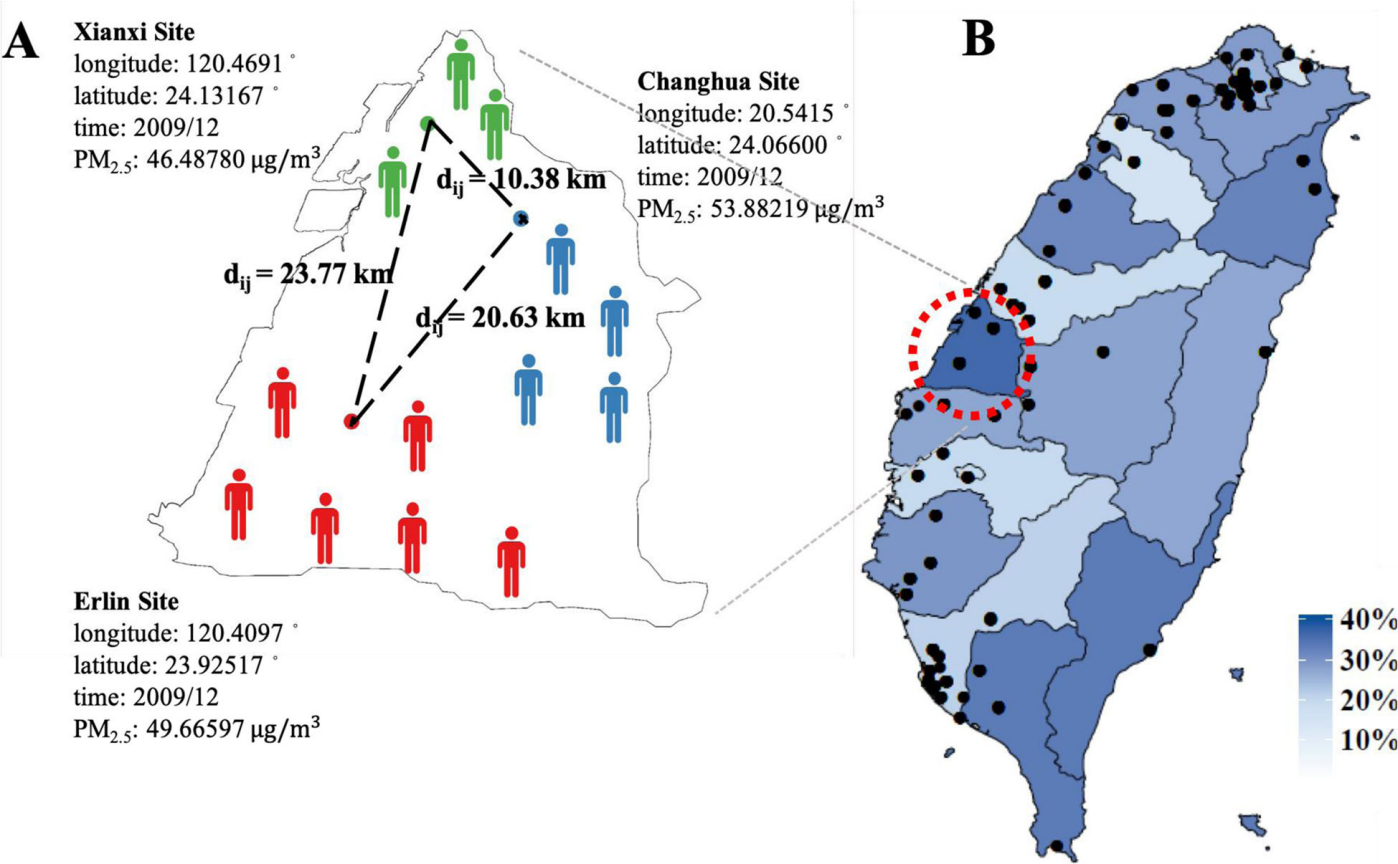
Determination of air pollution at individual level and spatial correlations and event rate of composite cardiovascular disease events and 76 air quality monitoring station (dots) across 20 geographic regions in Taiwan. **a** shows the two main problems inherent to existing studies on the environmental pollution effects on health. First, personal location is typically defined at the geographic area (community) level (e.g., a person is known to live in Changhua but their exact location in Changhua is unclear). As a result, the conventional approach for the assessment of air pollution is to assign all residents within an area the same value of air pollution, regardless of the individual’s relative location with respect to the pollution source. For example, consider the 13 people living in Changhua County shown in Fig. 1a. The average concentration for the three air monitoring stations within the area (i.e., [46.49+ 49.67 + 53.88]/3 = 50.01 μg/m^3^ for the monthly concentration of PM_2.5_ in Dec. 2009) is generally used for people living in a given area. Second, due to the coarse resolution used for individual locations, the distance between two individuals or their proxies (i.e., their assigned air quality monitoring stations) is not ascertained and thus the spatial correlations in the pattern of air pollution and adverse pollution effects on health are not modeled in the analyses. **b** shows the spatial variations in the event rate of cardiovascular diseases across Taiwan

Despite growing research on adverse health effects of environmental pollution, these studies typically face three major challenges. First, survival outcomes such as time to CVD events or death have become popular. However, applying classic analyses (e.g., Cox models) without specifying the spatial structure and considering possible spatial correlations in health and environmental data will likely underestimate adverse environmental pollution effects [16, 17]. Second, individual location is typically identified at the geographic area (e.g., community) level. Individual exposure to pollution is thus assessed using the average concentration of pollution within an area, with all residents assigned the same exposure concentrations [12, 17–19]. However, such an aggregated assessment of environmental pollution may be subjected to measurement errors, affecting the estimation precision of the adverse pollution effects on health outcomes. Exposure to air pollution may vary spatially for individual residents within an area, depending on the individual’s relative location with respect to the pollution source. Third, adverse pollution effects may also vary spatially across individuals, according to the distance between them. For example, the pattern of pollution exposure and consequently adverse pollution effects might be similar for individuals who are near each other. This is referred to as the spatial correlation between potential health effects and environmental hazards. However, due to the coarse geospatial resolution (e.g., broad geographic area) in previous studies, it is difficult to quantify the spatial correlation. In previous studies, spatial effects, including spatial correlation, are simply handled by using random effect models, where a variance term (e.g., marginal variances) is pre-defined but has no spatial structure or spatial correlation modeled in the analyses [17, 18, 20, 21]. The problems described above are also illustrated in Fig. 1a.

Against this background, we aim to assess the spatial association of ambient air pollutants with CVD risks in T2D patients, with the following advanced approaches: 1) health and environmental databases at the individual-level with longitudinal follow-ups are assembled to enrich study assessment, 2) a fine individual geospatial resolution (latitude and longitude) is determined to estimate the individual distance to the pollution source for measuring exposure to air pollution at the individual-level, and characterizing potential spatial correlation between environmental pollution and health outcomes, 3) a spatial structure/function is specified and the prior information is incorporated in a Bayesian spatial survival model as the main analysis, and 4) classic Cox and Weibull models are compared with the proposed Bayesian spatial survival model to demonstrate the improvement in the precision of estimating air pollution effects obtained using the rigorous Bayesian spatial analyses.

## Methods

### Data sources

We used national representative health claims data, Taiwan’s National Health Insurance Research Database (NHIRD), to derive a study cohort. We linked individual health claims data with Taiwan’s Environmental Protection Administration (EPA) air pollution data for a comprehensive assessment of the impact of ecological factors on health. Detailed geocoding for individual locations and data linkages are illustrated in Supplementary Figure 1 and addressed below.

### NHIRD

#### Brief description

The study population was identified from the NHIRD, which is derived from claims data of Taiwan’s National Health Insurance (NHI) program, a nationwide single-payment system that covers over 99% of Taiwan’s population. The NHIRD provides well-documented longitudinal data of medical diagnosis, procedure and utilization, laboratory testing, and prescription for enrolled individuals [22].

#### Study cohort and variables

To illustrate our approaches and corroborate our findings, we targeted T2D patients with high risks for CVDs (i.e., with diabetes duration of more than 8 years, under dual glucose-lowering agent [GLA] treatment, and with established CVDs) who are relatively homogenous regarding diabetes treatment course and vulnerable to environmental hazards for occurrence of CVDs [12]. Procedures of the study cohort extraction from the NHIRD are detailed in our previous studies [23–27]. Briefly, patients who were diagnosed with T2D and initiated with a second-line GLA added on to metformin were included. The initiation date of the second-line GLA was defined as the index date.

The primary health outcome during the follow-up was time to first hospitalized CVD event (a composite CVD with fatal/non-fatal events of myocardial infarction, ischemic heart disease, heart failure, ischemic and hemorrhagic stroke, cardiogenic shock, sudden cardiac arrest, arteriosclerotic cardiovascular disease, or arrhythmia) after the index date, using disease diagnosis and procedure codes listed in Supplementary Table 1. The accuracy of identifying CVD events in the NHIRD has been confirmed previously [28–30]. Each study patient was followed up from the index date until any CVD events occurred, dropout, or loss-to-follow-up from the NHI program, death, or the end of the database (i.e., 2013/12/31), whichever came first. Baseline patient characteristics (i.e., comorbidities, diabetes-related complications [31], CVD-related medications) were measured within 1 year before or at the index date.

#### Individual location determination based on a 5-step geospatial algorithm

A 5-step algorithm (as illustrated in Supplementary Figure 1) was used to identify the most frequently visited medical institutions (e.g., clinic for common medical problems, pharmacy for prescription refills) for determining individual locations, with three assumptions: 1) a T2D patient under GLA treatment usually went to the closest pharmacy for refilling their medications, 2) a patient generally visited the closest clinic or hospital for common medical problems such as a common cold, and 3) the township where the closest healthcare institution belonged to was then identified as an individual’s living environment, and the center of township at the level of longitude and latitude was assigned as the proxy for individual location; this proxy was applied because individual exact locations/addresses were de-identified in our study claims database for data protection of individual patients.

### Taiwan’s EPA data

#### Brief description

The air pollution information was collected from 76 monitoring stations over different geographic areas in Taiwan (shown as the dot points in Fig. 1b). The real-time concentrations of eight critical air pollutants, particulate matter (PM_2.5_, PM_10_), nitric oxide (NO, NO_x_), nitrogen dioxide (NO_2_), carbon monoxide (CO), sulfur dioxide (SO_2_), and ozone (O_3_), are routinely measured once every hour. The monthly averages of air pollutant concentrations from each air monitoring station were estimated in this study to represent the general pattern of local environmental pollution near the monitoring station.

#### Individual-level air pollution exposure

The exposure to air pollution for each individual was assigned, based on the data measured from the air quality monitoring station nearest the individual’s geographic location (using the center of township as proxy). Specifically, using latitude and longitude information of township’s center and air quality monitoring station, the nearest air quality monitoring station for each person was the station with the shortest Euclidean distance to the individual’s proxy location (township’s center). Hence, the air pollution and health claims data were linked at the individual-level based on the closest air quality monitoring station. The real-time concentrations of air pollutants for each individual were then represented as the median values of monthly concentrations of air pollutants during the follow-up. The median value was chosen because the median (compared to the mean) was less likely to be affected by the data variability measured over a relatively long period of time; in the other word, a median value was generally stable over time.

### Statistical modeling considerations

Our proposed statistical model was constructed based on classic survival analyses. The classic analysis approaches, the Cox and Weibull models, were first reviewed. The classic Cox model was chosen because it is commonly used in recent environmental studies with time-to-event data [12, 17, 19] and the Weibull model was selected because it is one of the most popular forms of parametric regression model [32]. Then, the proposed model with adjustment for spatial correlation based on Bayesian approaches was described. In our case study, the association between exposure to air pollutants and incident CVDs was estimated as the hazard ratio (HR) along with 95% credible interval (CrI) from our Bayesian model and 95% confidence interval (CI) from the Cox and Weibull models. Let *T* be the time from disease onset to an event of interest, and *X* denote a vector of baseline covariates with an arbitrary distribution. We assumed the proportional hazards model:

$$ \uplambda \left(\mathrm{t}|X\right)={\uplambda}_0(t)\exp \left( X\beta \right) $$

where *β* is a vector of parameters and λ_0_(*t*) is an unspecified baseline hazard function. An example for the baseline hazard function is the Weibull baseline hazard, λ_0_(*t*) = *λt*^*α*^, with parameters *λ* > 0 and α > 0, which is one of the popular forms of parametric regression model.

To incorporate spatial information, we applied the model proposed by Taylor and Barry [33]:

$$ \lambda \left(\mathrm{t}|X\right)={\lambda}_0(t)\exp \left( X\beta +Y\right) $$

where *Y* is added to the model and explain as a set of a spatially continuous, stationary latent Gaussian field at the location of each observation. In general, exp(*Y*) can be directly interpreted as a multiplicative scaling on the hazard function. One common choice to account for spatial structure is a distance-based exponential covariance function, while many other options for spatial correlation are available [34]. We assumed the exponential covariance function for *Y*, *Cov*(*Y*_*i*_, *Y*_*j*_) = *σ*^2^ exp(−*d*_*ij*_/*ϕ*), where *d*_*ij*_ represents the Euclidean distance between the coordinates of the *i*^*th*^ observation and those of the *j*^*th*^ observation (i.e., two individuals or their proxies such as their assigned air quality monitoring stations; Fig. 1a), *σ*^2^ is the marginal variance of the latent field, and *ϕ* represents the spatial decay parameter (larger values indicate a longer range of spatial dependence).

These probability distributions depend on unknown parameters *β*, *λ*, *α*, *σ*, and *ϕ*. In the Bayesian framework, the model parameters are expressed by placing a probability distribution on the parameters, called the prior distribution, which represents one’s beliefs about this quantity when pilot dataset is considered. After a dataset conditional on the uncertain quantity is collected, we have the posterior probability distribution, which is the probability distribution of an unknown quantity conditional on the current evidence. With an appropriate choice for the prior information, it is possible to use Markov chain Monte Carlo methods [35, 36] to draw samples from the posterior density and hence perform Bayesian inference. Statistical model-checking was performed and a model with a smaller deviance information criterion was considered satisfactory. In Supplementary description, we provided more mathematical details of the choice of prior distributions for each parameter.

For Bayesian spatial survival model building, we used the package “spatsurv” and created a 95% CrI, which indicates that there is a 95% chance that the true value lies within the middle region, by taking the 2.5 and 97.5% percentile points of this posterior distribution. The analyses were performed using statistical software R 3.4.2.

## Results

Figure 1b shows the event rate of composite CVD events across 20 geographic regions in Taiwan, with the highest rate in Changhua (36.1%) and the lowest rate in Keelung (14.7%). The Kaplan-Meier curve for composite CVD events is presented in Supplementary Figure 2, with a censoring rate of 73.8% from a total of 2072 study patients with 542 CVD events occurring during the follow-up.

Table 1 shows the descriptive results of characteristics of study patients and univariate analysis results for the composite CVD risks associated with individual patient characteristics based on our Bayesian spatial survival model. Significant associations (HR [95% CrI]) were observed for demographics (male: 1.220 [1.022–1.474], age: 1.036 [1.028–1.045]), diabetes-related status (e.g., adapted Diabetes Complication Severity index [aDCSI]: 1.211 [1.170–1.255]), GLA use (e.g., DPP-4i: 0.670 [0.514–0.942]), comorbidities (e.g., hypertension: 1.279 [1.031–1.595]), CVD-related medications (e.g., *β*-blocker: 1.172 [1.005–1.343]), and several air pollutants, including CO (3.791 [1.945–8.088]), NO (1.088 [1.032–1.129]), NO_2_ (1.120 [1.079–1.166]), NO_x_ (1.066 [1.046–1.093]), PM_10_ (1.052 [1.028–1.070]), PM_2.5_ (1.105 [1.080–1.135]), and SO_2_ (2.095 [1.738–2.352]).

**Table 1:** Characteristics of study patients and their associations with developing composite cardiovascular disease events based on the results of univariate analyses of Bayesian spatial survival analysis

Characteristics	Mean (SD) or proportion	HR (95% credible interval)
Age at index date (years)	69.573 (10.939)	1.036 (1.028, 1.045)
Male (%)	52.075%	1.220 (1.022, 1.474)
Duration of diabetes at index date (years)	9.647 (1.112)	0.935 (0.867, 1.040)
aDCSI (score)	2.296 (1.856)	1.211 (1.170, 1.255)
Second-line glucose-lowering agent at index date (%)		
Sulfonylurea	65.347%	(ref.)
Acarbose	10.907%	0.921 (0.682, 1.192)
Dipeptidyl-peptidase 4 inhibitor	10.714%	0.670 (0.514, 0.942)
Meglitinide	8.639%	1.733 (1.436, 2.170)
Thiazolidinedione	4.393%	1.116 (0.713, 1.703)
Comorbidities and diabetes-related complications within 1 year before index date (%)		
Hypertension	77.703%	1.279 (1.031, 1.595)
Dyslipidemia	42.568%	0.786 (0.647, 0.908)
Retinopathy	32.288%	1.142 (0.971, 1.321)
Nephropathy	13.851%	1.392 (1.056, 1.703)
Neuropathy	17.905%	0.899 (0.710, 1.079)
Peripheral vascular disease	9.846%	1.301 (0.968, 1.658)
CVD-related medications within 1 year before index date (%)		
Lipid-modifying agent	43.243%	0.939 (0.781, 1.112)
α-blocker	8.929%	1.090 (0.806, 1.445)
β-blocker	48.069%	1.172 (1.005, 1.343)
Renin-angiotensin-aldosterone agent	52.027%	1.194 (0.973, 1.408)
Diuretics	28.427%	1.941 (1.638, 2.282)
Calcium channel blocker	58.832%	1.074 (0.912, 1.259)
Antiarrhythmic	6.129%	1.459 (1.131, 1.822)
Cardiac glycoside	4.778%	2.962 (2.277, 3.859)
Vasodilator	29.054%	2.352 (2.000, 2.742)
Antiplatelet agent	69.932%	1.166 (0.984, 1.397)
Anticoagulant	2.799%	2.104 (1.468, 3.298)
Monthly concentrations of air pollutants during the follow-up		
CO (ppm)	0.501 (0.170)	3.791 (1.945, 8.088)
NO (ppb)	5.446 (4.026)	1.088 (1.032, 1.129)
NO_2_ (ppb)	17.320 (5.628)	1.120 (1.079, 1.166)
NOx (ppb)	22.603 (8.993)	1.066 (1.046, 1.093)
O_3_ (ppb)	27.767 (4.648)	1.024 (0.984, 1.073)
PM_10_ (μg/m^3^)	54.506 (17.207)	1.052 (1.028, 1.070)
PM_2.5_ (μg/m^3^)	31.294 (9.425)	1.105 (1.080, 1.135)
SO_2_ (ppb)	3.876 (1.527)	2.095 (1.738, 2.352)

*Abbreviations*: *SD* Standard deviation, *aDCSI* adapted Diabetes Complication Severity Index, *CVD* Cardiovascular disease

Index date refers to the date of the beginning of second-line glucose-lowering agent therapy

**Fig. 2:**
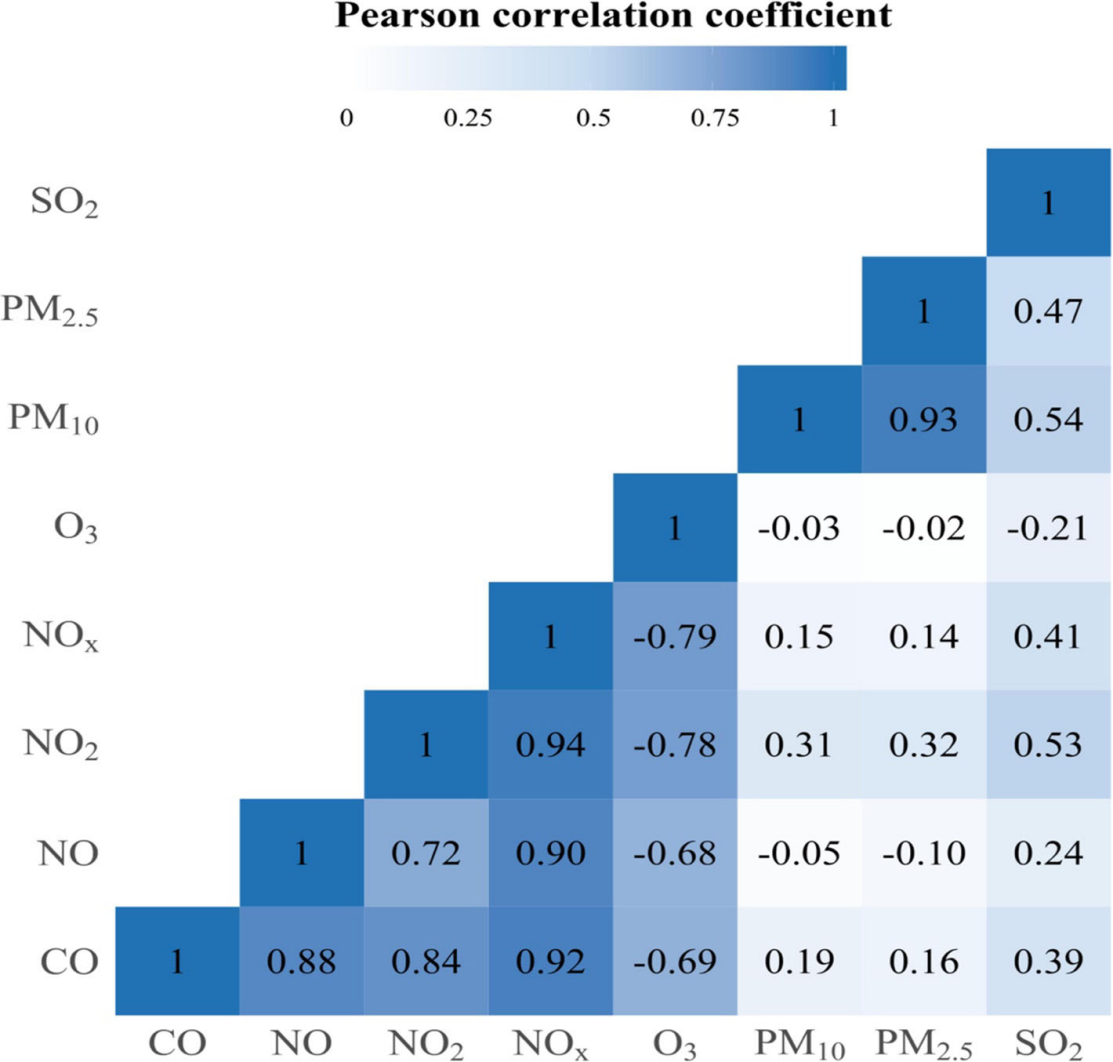
Collinearity between ambient air pollutants tested using Pearson correlation matrix. This figure shows that several ambient air pollutants are highly correlated to each other (e.g., there are correlations between PM_10_ and PM_2.5_ [i.e., 0.93] and between NO_x_ and NO_2_ [i.e., 0.94])

According to a heat map from the Pearson correlation matrix shown in Fig. 2, we only selected NO, PM_2.5_, and SO_2_ in the final multivariate model, in order to avoid the multicollinearity problem (e.g., a high correlation between PM_10_ and PM_2.5_; 0.93).

**Fig. 3:**
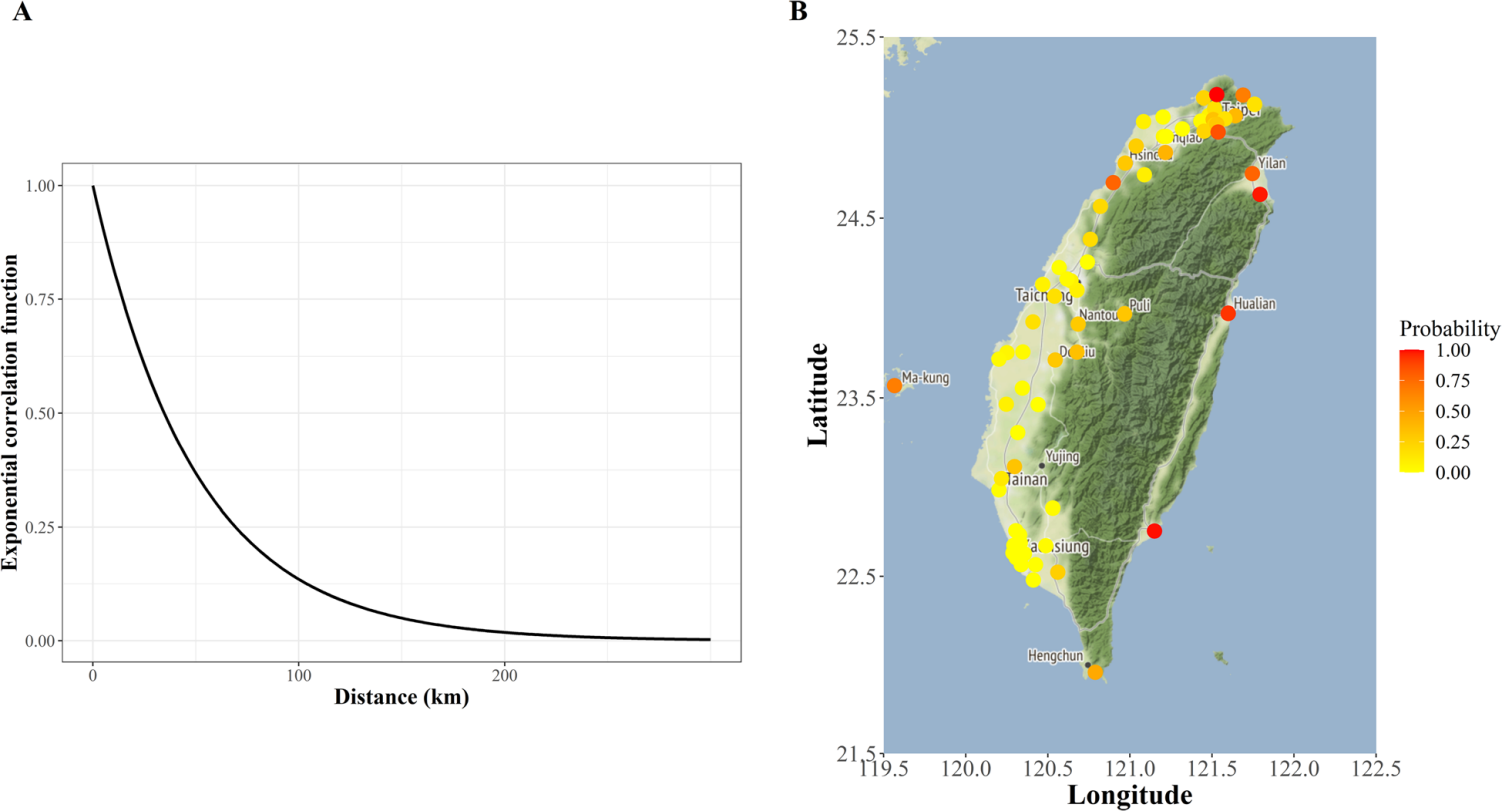
Estimated spatial correlations and predicted risk-exceedance probabilities (P [exp(Y) > 1]) of cardiovascular diseases in Taiwan. **a** Estimated spatial correlations of exposure to air pollution and consequent pollution effects on cardiovascular diseases. Based on the estimated spatial parameters *σ* = 1.28 and *ϕ* = 0.44, when the distance between the proxies for two individuals (i.e., two air quality monitoring stations assigned to them) is more than 100 km, the corresponding relative spatial correlations with the hazard of CVDs between these two individuals reduce to almost zero; this means the spatial variability that cannot be explained by the covariates in the model is almost zero when two stations are located more than 100 km apart from each other. **b** Predicted risk-exceedance probabilities (P [exp(Y) > 1]) of cardiovascular diseases for individual geographic areas in Taiwan. This plot shows the posterior probability that the covariate-adjusted relative risk of CVDs is larger than 1 (P [exp(Y) > 1]), which maps the likelihood of excessive CVD risks as the “CVD risk-exceedance probability” to demonstrate the risk level of CVDs for areas in Taiwan. Specifically, for the areas with a color spot close to red that indicates the likelihood of having CVDs (i.e., covariate-adjusted relative risk > 1) is close to 100%, they could be classified as the area with a higher CVD risk. In contrast, for those with a color spot close to yellow that implies the likelihood of having CVDs > 1 is close to 0%, they could be considered as the area with a lower CVD risk

Figure 3a shows the estimated exponential spatial correlation function for the pattern of air pollution and its adverse effects on CVDs. Based on the estimated spatial parameters *σ* = 1.28 and *ϕ* = 0.44, when the distance between the proxies for two individuals (i.e., two air quality monitoring stations assigned to them) is more than 100 km, the corresponding relative spatial correlations with the hazard of CVDs between these two individuals reduce to almost zero; this means the spatial variability that cannot be explained by the covariates in the model is almost zero when two stations are located more than 100 km apart from each other. Figure 3b then shows the posterior probabilities of the covariate-adjusted relative risks of CVDs for individual geographic areas in Taiwan; the color spots close to red indicate the corresponding areas at higher CVD risks, while the color spots close to yellow suggest the areas with lower CVD risks.

**Fig. 4:**
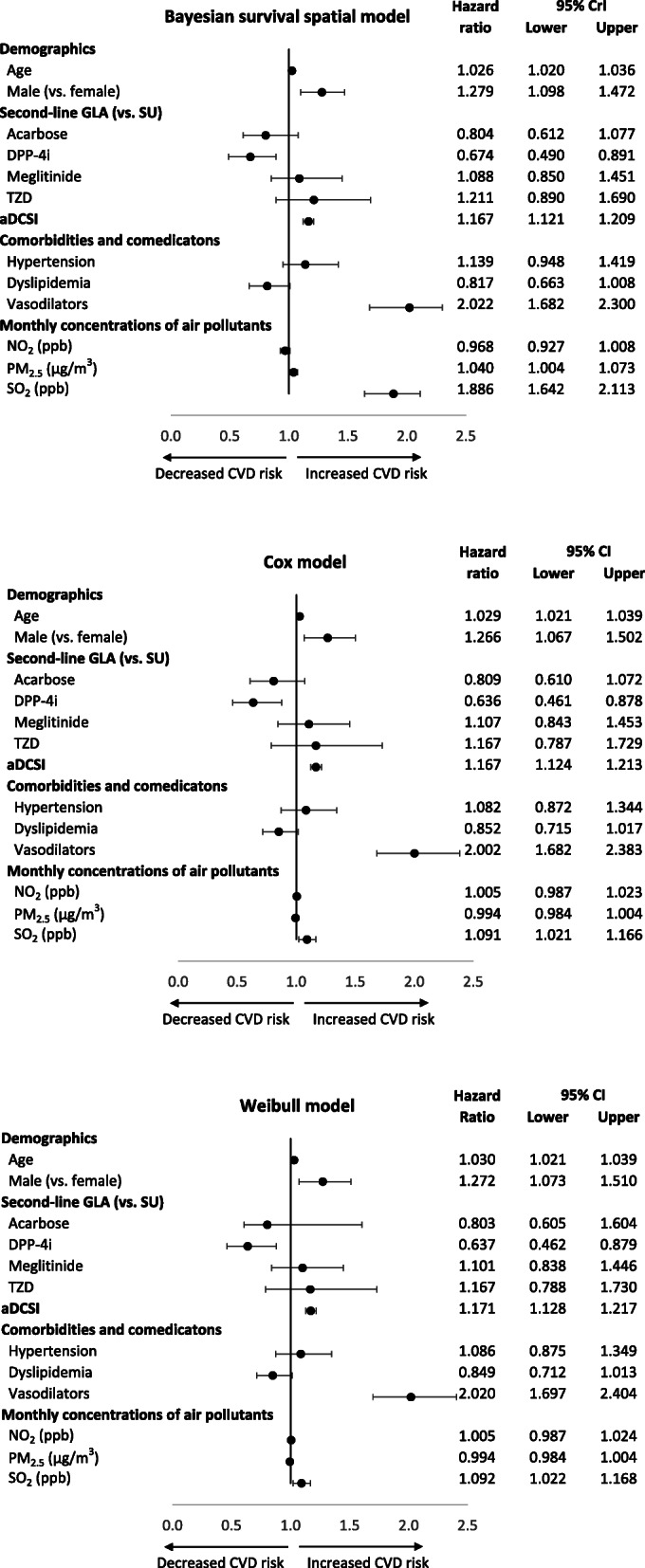
Association between patients’ characteristics and the risk of cardiovascular diseases in the multivariable Bayesian survival spatial, Cox, and Weibull models. This figure shows the association between patients’ characteristics (i.e., demographics, drug treatments, diabetes status/severity, comorbidities, and air pollution exposure) and the risk of composite cardiovascular disease events, estimated by the multivariable Bayesian survival spatial, Cox, and Weibull models. And, in the Bayesian survival spatial model, the estimated prior parameters *α, λ, σ,* and *φ* are 0.932, 0.0001, 1.2829, and 0.440, respectively. For the Weibull model, *α* and *λ* are 0.8971 and 0.0007, respectively. Abbreviations: aDCSI, adapted Diabetes Complication Severity Index; CVD, cardiovascular disease; GLA, glucose-lowering agent; SU, sulfonylurea; DPP-4i, dipeptidyl-peptidase 4 inhibitor; TZD, thiazolidinedione; CrI, credible interval; CI, confidence interval

Figure 4 shows the results of three final multivariate models (i.e., our Bayesian spatial survival, the classic Cox and parametric Weibull models) for the association between air pollutants and CVD risks, with adjusted for other significant patient characteristics shown in the univariate analyses (i.e., Table 1). Across the three models, being male, aDCSI score, and use of DPP-4i or a vasodilator are statistically significantly associated with CVD risks, while age at initiation of the second-line GLA had a borderline significant association with CVD risks. The associated coefficients and significance levels for these variables are similar across the three models.

However, the results on adverse CVD effects of air pollutants are different across the three models; in our Bayesian spatial survival model, PM_2.5_ is a significant risk factor for CVDs (the adjusted HR [95% CrI] for a 1 μg/m^3^ increase in the monthly PM_2.5_ concentration is 1.040 [1.004–1.073]), but it was not in the Cox or Weibull models (0.994 [0.984–1.004]). Also, SO_2_ is a significant risk factor across the three models, but the magnitude of its adverse CVD effect in our model is the largest; with a 1 ppb increase in the monthly SO_2_ concentration, the adjusted HR (95% CrI or CI) is 1.886 (1.642–2.113), 1.091 (1.021–1.166), and 1.092 (1.022–1.168) from the Bayesian spatial survival, Cox, and Weibull models, respectively.

## Discussion

This is the first study to apply Bayesian spatial survival approaches, with the fine-resolution of individual locations and the specifications of spatial structure and exponential correlation function for analyzing environmental pollution effects on CVD risks among a T2D population. Significant adverse health effects of environmental pollution among vulnerable T2D patients were confirmed using our approaches, whereas these deleterious effects were attenuated in the classic modeling approaches (Cox and Weibull models). This supports the validity of our approaches against the traditional analyses. This study therefore provides more suitable and precise methodologies for studying spatial survival data, urges more awareness of ecological risk factors to health, and supports the development of spatiality-oriented management strategies to minimize or avoid adverse environmental pollution effects.

Our approaches have several strengths. First, compared to existing studies where individual locations are commonly defined at the geographic area level (as spatially discrete variable) and the average air pollution concentrations within an area are typically assigned for all people living in that area, we adopted a finer location resolution for each study patient’s location using the center of township as proxy at the level of latitude and longitude (as spatially continuous variable), which facilitates both the determination of individual-level environmental pollution exposure and the estimation of the spatial correlation with health data (*d*_*ij*_), and therefore enhances the measurement precision. Second, we specified the spatial structure with an embedded exponential correlation function, and applied Bayesian approaches that allow appropriate prior information to be utilized, to provide more information for parameter estimation and mitigate the potential problem of measurement error. Third, individual-linked longitudinal data between health claims (i.e., NHIRD) and ecological data (i.e., EPA data) were utilized, to enhance the comprehensiveness of assessing the health impact of environmental hazards.

Our results confirm the significant adverse cardiovascular effects of environmental pollutants (i.e., PM_2.5_, SO_2_) among vulnerable T2D patients with high CVD risks. However, the significance of adverse CVD risks associated with exposure to PM_2.5_ was close to null in the classic Cox and Weibull models. The harmful effect of PM_2.5_ on cardiovascular outcomes has been confirmed in previous studies [6, 9], and our study vulnerable T2D patients were previously shown to be sensitive to adverse effects of air pollution [9, 12, 13]. This means that the classic models may have underestimated adverse health effects due to exposure to PM_2.5_. Also, ample evidence [37–41] supports that exposure to SO_2_ increases CVD risks by up to around 30% (i.e., 1.94 [1.78–2.11] for a 2.54 ppb increase of SO_2_ [41]) among the general population. And, the higher estimate of the CVD risk associated with exposure to SO_2_ shown in our model versus that from the Cox or Weibull model can be supported as follows. First, compared to other countries, SO_2_ concentration is higher in Taiwan due to the high numbers of coal-fired power plants, scooters, and cars in this country [40]. Second, since our study population was the T2D patients with high CVD risks (e.g., age > 60 years, with comorbid hypertension or dyslipidemia condition [42]), they might be more vulnerable to adverse effects associated with exposure to SO_2_.

Moreover, one should note that variations in statistical results across the three models were only observed in the variables of ambient air pollutants (i.e., Fig. 4). The statistical results for associations between traditional risk factors and CVD risks were similar across the three models, which are comparable with the previous study results (i.e., age [43], gender [43], diabetes-related complications [44, 45], use of GLAs [23, 24]). This implies that, for the variables with no or limited spatial variations (i.e., traditional risk factors), a spatial-driven model such as our model will yield results similar to those obtained with non-spatial based models (i.e., Cox or Weibull model). In this case, no statistical model is preferred. However, for the factors with spatial variations (i.e., ambient air pollutants), it is important to utilize our Bayesian spatial analyses because the classic modeling analyses may not be able to handle spatial effects.

There are several limitations inherent to our approaches. First, we did not differentiate between indoor and outdoor air microenvironments. However, most people are outside for a small fraction of the time and the amount of air pollution that penetrates indoor environments might be modified by building characteristics. Outdoor measurements are thus of limited value for estimating the actual exposure of humans to many airborne contaminants. Second, a person’s living area (in terms of township in the present study) and associated air pollution may change over time. These time-dependent/varying issues were not considered in our analyses because the Bayesian spatial survival model was not developed to handle time-varying data. This suggests a need for future research to include space-time dynamics in the model estimation (e.g., time-varying models). Alternatively, modeling multiple township as proportional to the frequency of each township that occurred in all individual’s visited townships over follow-up time in analysis could be another approach to deal with such a time-dependent issue. This is another direction deserved for future research. Third, data of each individual household location in our claims was unavailable due to protection of personal data and privacy. The potential problem of spatial misalignment for an individual location may occur when a person’s location was defined using the 5-step geospatial algorithm in this study. However, this problem may less affect this study with the following reasons. 1) The township of the healthcare institution that a person’s most visited for common medical services could be considered as a person’s daily living environment. The individual’s daily living environment, instead of the exact household location, was the main interest of this study, and then the air pollution data provided by the air quality monitoring stations within that living environment (i.e., township) were assigned to the individual. This would be expected to provide more comprehensively spatial assessment for the impact of air pollution on a patient’s health. 2) The center of township was used as the proxy for individual location. Since the average area of township in Taiwan is relatively small (ranging from 28.8 to 4628.6 km^2^) and any two air quality monitoring stations within a township is generally close (the mean, minimum and maximum distances: 10.3, 0.04 and 54.4 km, respectively), the problem of spatial misalignment for individual location within a township would have less effect to our study results. 3) If spatial misalignment occurs due to spatial aggregation problems, our Bayesian approaches with probability formulas and prior information on the potential measurement error probabilities of outcomes were expected to mitigate this issue [46]. Nevertheless, to understand the potential problem of spatial misalignment in this study, future studies are warranted to model the individual’s exact house location in analysis. Lastly, the generalizability of study results may be limited to a population with T2D and high risks for CVDs.

## Conclusions

We applied the Bayesian spatial survival model as an extension of classic analysis procedures, utilized finer individual geo-resolution (latitude, longitude), specified a spatial structure, and modeled spatial correlation, to extend the existing knowledge of significant adverse cardiovascular effects of environmental pollution from the general population to vulnerable diabetic patients. Adverse cardiovascular effects of air pollution among T2D patients should be recognized to support the development of spatiality-oriented management strategies to minimize or avoid adverse environmental pollution effects. And, our rigorous methodologies should be promoted to environmental research with time-to-event data to enhance the precision of study assessment and ensure the validity of study results.

## Supplementary information

**Additional file 1.**

## Data Availability

The datasets generated and/or analyzed during the current study are not publicly available due to data privacy issues but are available from the corresponding author on reasonable request.
