# Supplementary material for: Association of ambient air pollution with cardiovascular disease risks in people with type 2 diabetes: a Bayesian spatial survival analysis
Source: Environ Health. 2020 Nov 5;19:110. doi: 10.1186/s12940-020-00664-0 (PMC7643356; doi:10.1186/s12940-020-00664-0)

**Supplementary Figure 1. Determination of individual geo-location and linkage between health claims data from the National Health Insurance Research Database (NHIRD) and pollution data from the Environmental Protection Administration (EPA) in Taiwan**


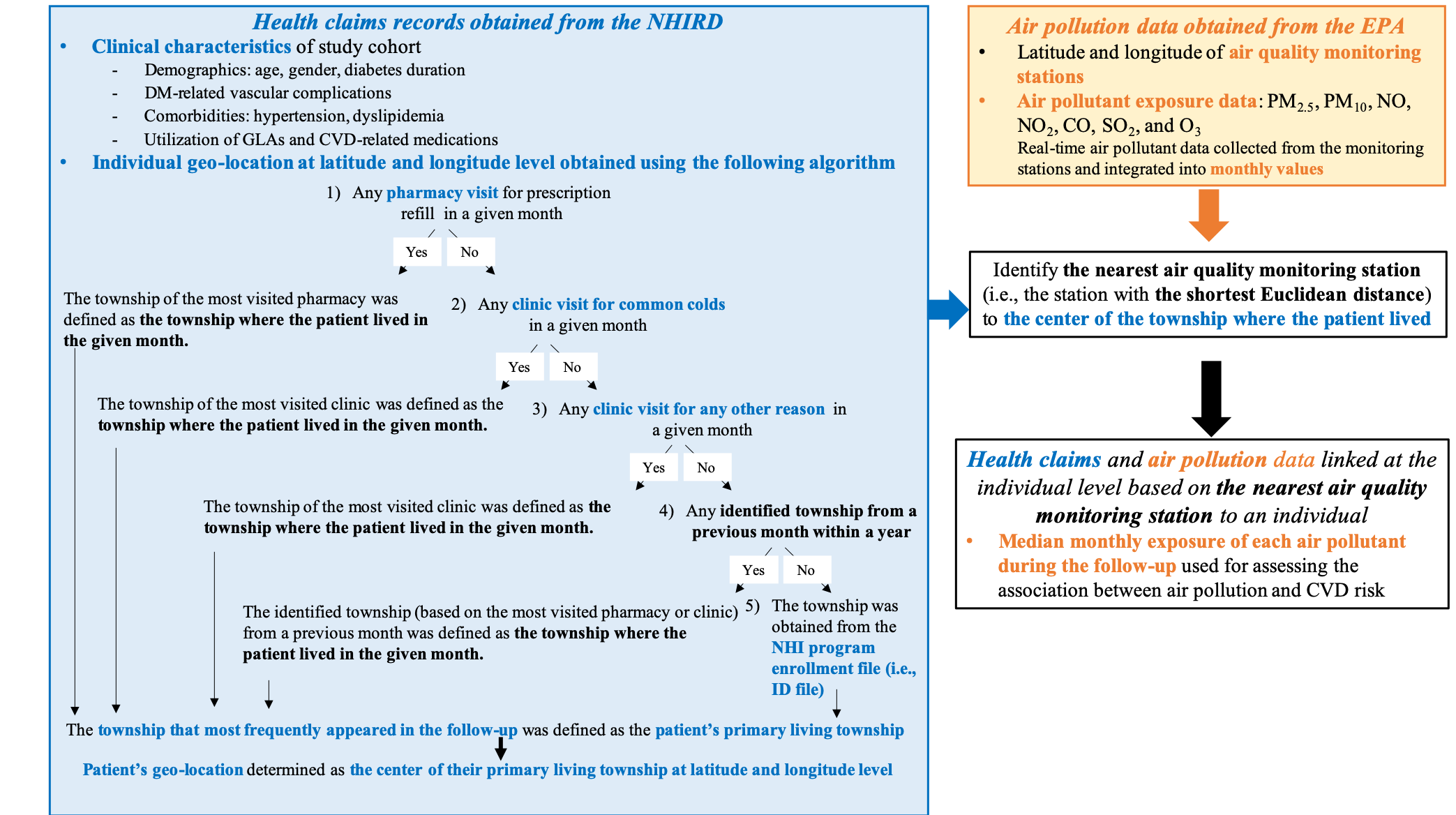


Abbreviations: GLAs: glucose-lowering agents, CVD: cardiovascular disease.

**Legend:**

Although the enrollment records in the NHIRD indicate the geographic location information for each beneficiary, the data are based on the location (i.e., township) of the insured unit/group, which might not reflect the most current residential location of an individual. Therefore, a 5-step procedure was applied to define the nearest medical institution to where a patient lived in each month during the follow-up period include: 1) if the patient had visited any pharmacies for chronic prescription refills for medications (e.g., GLAs) in a given month, the most frequently visited pharmacy was regarded as the nearest medical institution near the patient’s living area; 2) if the patient had never visited a pharmacy but had a record of a clinic visit for a common cold in a given month, the most frequently visited clinic was regarded as the nearest medical institution near the patient’s living area; 3) if the patient had never visited a pharmacy or clinic for a common cold in a given month, the clinic with the most visits for any reason was regarded as the nearest medical institution near the patient’s living area; 4) if the patient had not visited a pharmacy or clinic in a given month but had a record for criterion 1), 2), or 3) in a previous month within a year, then the location defined for this criterion for the most recent month with a record was used as the patient’s living area, and 5) if none of the above criteria were met, the geographic location recorded in the NHIRD enrollment file was used as the place where the patient lived.

The township for the medical institution (i.e., pharmacy, clinic) or geographic location (i.e., from the NHIRD enrollment file) determined above was then assigned as the township where a patient lived. The township which most frequently appeared during the follow-up for each individual was assigned as the primary township. The center of the primary township (at the latitude and longitude level) was defined as the individual’s geo-location in the analyses.

**Supplementary Table 1. International Classification of Disease, 9^th^ Edition, Clinical Modification (ICD-9-CM) codes used to define cardiovascular diseases**

| Cardiovascular diseases | ICD-9-CM disease codes | ICD-9-CM procedure codes |
| --- | --- | --- |
| Myocardial infarction | 410 | --- |
| Cerebrovascular diseases | 430-427, V12.54 | 38.11, 38.12 |
| Heart failure | 428 | --- |
| Ischemic heart diseases | 411, 413, 414, V45.81, V45.82 | 00.66, 36.0-36.3, 36.9, 88.5 |
| Aortic aneurysm/dissection | 441 | --- |
| Cardiogenic shock | 785.51 | --- |
| Sudden cardiac arrest | V12.53 | --- |
| Arteriosclerotic cardiovascular diseases | 429.2 | --- |
| Arrhythmia | 426, 427 | --- |

**Supplementary Description. Bayesian framework in this study**

The estimation depended on the prior distribution of parameters $\beta, \lambda, \alpha, \sigma, and \phi$ using Bayesian approaches. Empirical Bayes methods were used for statistical inference, in which the prior distribution was estimated from the data of our study cohort. To incorporate spatial information, we applied

$$\lambda\left( t | X \right)=\lambda_{0}(t,w)exp(X\beta+Y)$$

with the parameter $\beta$, which was expressed by a prior distribution as:

$\beta\sim N({0,3}^{2})$.

Because the baseline hazard $\lambda_{0}\left( t \right)=\lambda t^{\alpha}$ and $\lambda$ and $\alpha$should be positive, we assumed the following distributions based on the information from our data:

$log(\lambda)\sim N({0,3}^{2})$,

$log(\alpha)\sim N({0,3}^{2})$.

As estimated using the R package geoR,^1^ the prior distributions of$\sigma$and$\phi$ were respectively specified as:

$\log\left( \phi\right)\sim N(0, 1)$,

$\log\left( \sigma\right)\sim N(0, 1)$.

The initial estimates of parameters$\beta$, $\lambda$, and$\alpha$can be obtained via the maximum likelihood method, where any spatial correlations between observations are typically ignored.

After a dataset conditional on the uncertain quantity was collected, we had the posterior probability distribution, which showed the probability distribution of an unknown quantity conditional on the current evidence from our data.

It is therefore possible to use Markov chain Monte Carlo (MCMC) methods^2,3^ to draw samples from the posterior density and hence perform Bayesian inference.

In this study, the MCMC chain was run for 22,000 iterations with a 2,000 iteration burn-in, retaining every 100^th^ sample.^4^

**References:**

1. Ribeiro Jr PJ, Diggle PJ. geoR: Package for Geostatistical Data Analysis an illustrative session. *Artificial Intelligence.* 2006;1:1-24.

2. Metropolis N, Rosenbluth AW, Rosenbluth MN, Teller AH, Teller E. Equation of state calculations by fast computing machines. *The journal of chemical physics.* 1953;21(6):1087-1092.

3. Hastings WK. Monte Carlo sampling methods using Markov chains and their applications. 1970.

4. Taylor BM. Auxiliary variable Markov chain Monte Carlo for spatial survival and geostatistical models. *arXiv preprint arXiv:150101665.* 2015.

**Supplementary Figure 2. Survival curve for composite cardiovascular disease events of study cohort**


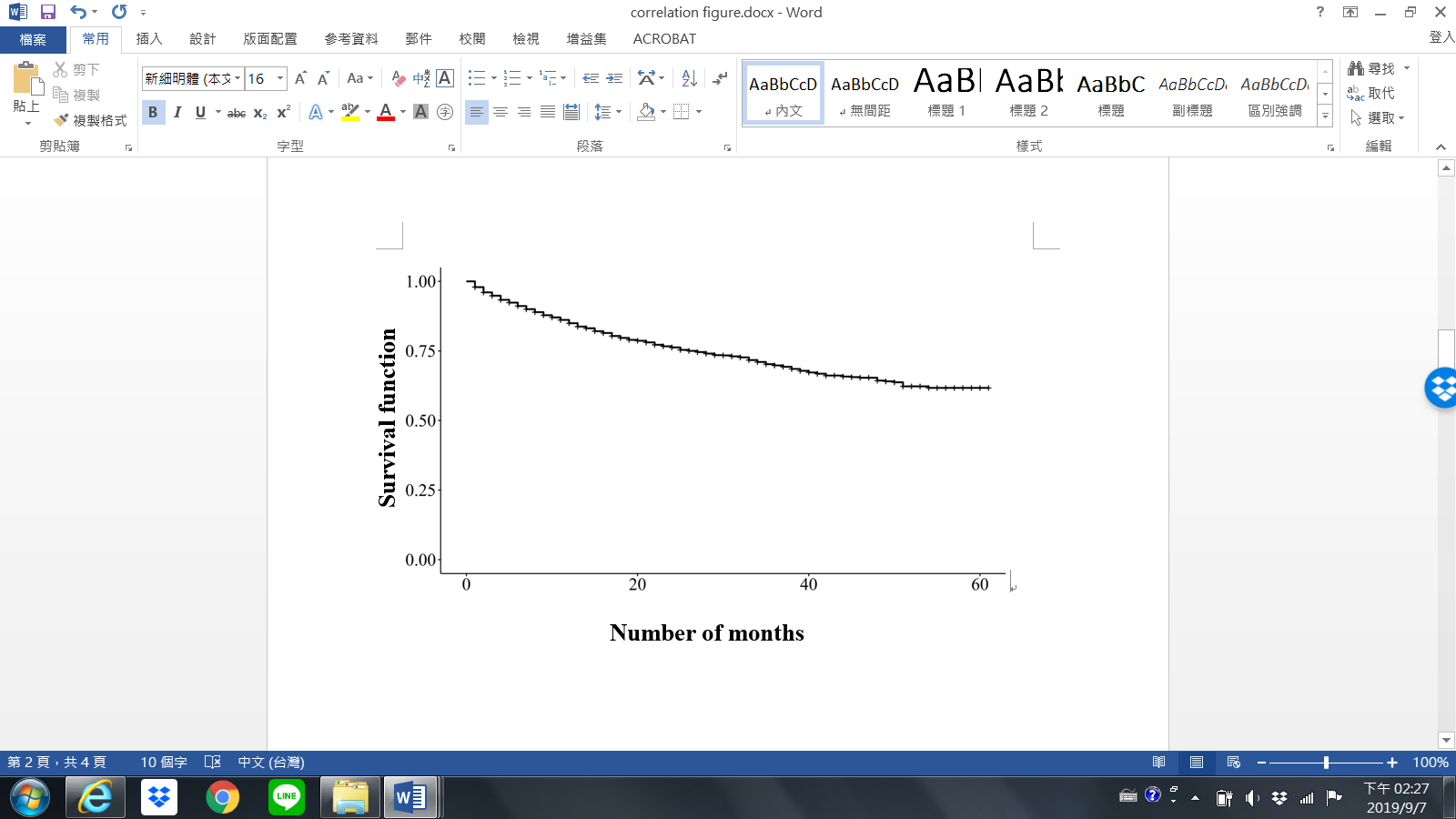

Supplement: Supplementary file 1 — Additional file 1. [file 12940_2020_664_MOESM1_ESM.docx]
